# Training needs assessment of European healthcare workers on vaccinology and vaccine acceptance: Andrea Paladini

**DOI:** 10.1093/eurpub/ckac130.151

**Published:** 2022-10-25

**Authors:** A Paladini, TE Lanza, V Gianfredi, L Blandi, W Ricciardi, G Damiani, C Signorelli, A Odone, C Cadeddu

**Affiliations:** 1 Life Sciences and Public Health, Università Cattolica del Sacro Cuore, Rome, Italy; 2 School of Medicine, Vita-Salute San Raffaele University, Milan, Italy; 3 Public Health, University of Pavia, Pavia, Italy

## Abstract

**Background:**

Healthcare workers (HCWs) are at the frontline of interaction with those that are taking decisions around vaccination. They need adequate training. The general aim of this systematic review is to assess HCWs’ training needs on vaccinology and vaccine acceptance. This work was performed for the European Centre for Disease Control (ECDC) under the specific contract No 1ECD.12108 ID.12922 implementing the framework contract number ECDC/2021/005.

**Methods:**

The search was performed using MEDLINE, Scopus and Google Scholar databases in February 2022. The following inclusion criteria were used: date (from 01/01/2011 to 24/02/2022); language (English, Italian, Portuguese, Spanish, French and Romanian); geographic location of the study (Europe). Appraisal tool for Cross-Sectional Studies (AXIS checklist) was used to assess the methodological quality of the included papers.

**Results:**

The scientific literature search retrieved 640 results on PubMed, 556 on Scopus and 15 on Google Scholar. In total, 1211 records were identified. After the duplicate removal and the title/abstract assessment, 132 publications were assessed for eligibility. Finally, after the full-text assessment, only 25 articles were included. As regards for the quality assessment, all studies were judged of moderate-good quality. The majority of studies stressed the need to deepen general knowledge of vaccine preventable diseases, vaccine efficacy, vaccination schedule and adverse effects of vaccines.

**Conclusions:**

Considering their role in the community, especially as source of information and trust for vaccine acceptance, educational initiatives in vaccinology and vaccine hesitancy should be prioritized for HCWs, aimed at increasing their knowledge, awareness, and attitudes. An important heterogeneity of educational backgrounds, activities performed and training needs of the HCWs involved in vaccination at European level was one of the main critical issue to be addressed for future actions.

**Key messages:**

- HCWs need appropriate training and constant updates on immunization.
- General knowledge of vaccine preventable diseases, vaccine efficacy, vaccination schedule and safety are main HCWs’ learning needs.

